# Photocontrol of bacterial membrane potential regulates antibiotic persistence in *B. subtilis*

**DOI:** 10.1140/epjp/s13360-025-06263-7

**Published:** 2025-04-24

**Authors:** Pietro Bertolotti, Federico Gallinardi, Marta Ghidoli, Chiara Bertarelli, Guglielmo Lanzani, Giuseppe Maria Paternò

**Affiliations:** 1 Center for Nanoscience and Technology, Istituto Italiano di Tecnologia, Via Rubattino 81, 20134 Milan, Italy; 2 Department of Electronics, Information and Bioengineering, Politecnico di Milano, Via Ponzio 34/5, 20133 Milan, Italy; 3 Department of Biotechnology and Bioscience, Università di Milano – Bicocca, Building U3 – BIOS, Piazza della Scienza 2, 20126 Milan, Italy; 4 Department of Chemistry, Materials and Chemical Engineering, “Giulio Natta” Politecnico di Milano, Piazza Leonardo Da Vinci 32, 20133 Milan, Italy; 5 Department of Physics, Politecnico di Milano, Piazza Leonardo Da Vinci 32, 20133 Milan, Italy

## Abstract

Bacterial persistence and resistance to antibiotics pose critical challenges in healthcare and environmental contexts. Recent studies revealing that bacteria possess a dynamic electrical membrane potential open new avenues for influencing bacterial behaviour and drug susceptibility. In this work, we present a novel light-responsive strategy to modulate bacterial antibiotic persistence using Ziapin2, an azobenzene photoswitch previously shown to alter bacterial membrane potential. We selected two broad-spectrum antibiotics with distinct modes of action: Kanamycin, which requires cytosolic uptake to inhibit protein synthesis, and Ampicillin, which targets cell wall polymerization at the cell envelope, to probe the role of membrane potential in antibiotic efficacy. Our findings show that when *Bacillus subtilis* is exposed to Kanamycin and Ziapin2, photoactivation (470 nm) significantly impacts bacterial viability: under illumination, the previously lethal effects of Kanamycin are markedly reduced, suggesting that membrane potential changes drive altered antibiotic uptake or intracellular accumulation. In contrast, Ampicillin-treated samples remain largely unaffected by light-induced membrane modulation, consistent with its action at the external cell envelope. Taken together, these results indicate that membrane potential manipulation can selectively influence the activity of antibiotics whose intracellular uptake is critical to their function. This proof-of-concept study underscores the potential of non-genetic, light-based interventions to modulate bacterial susceptibility in real time. Future work will expand this approach by exploring additional antibiotic classes and novel azobenzene derivatives, ultimately advancing our understanding of bacterial bioelectric regulation and its applications in antimicrobial therapies.

## Introduction

The study and characterization of the interaction between bacterial cells and antibiotics to investigate their mechanisms of tolerance and resistance is of increasing importance. The induction of various forms of resistance to major broad-spectrum antibiotics, in fact, constitutes a primary issue in many sectors, notably healthcare and environment [1, 2]. However, while resistance is a phenomenon which involves a change in an organism’s genome, persistence is usually the first step that leads to resistance [3], which consists of the intrinsic capability of a cellular population to tolerate a certain limited amount of antibiotic. A prolonged persistence under selective pressure can induce to random genetic mutations eventually leading to resistance.

In recent years, it has been discovered that, similarly to what was observed in excitable eukaryotic cells, bacteria also possess a dynamic membrane potential [4–7], which can be modulated by external stimuli [8–13]. This potential is primarily involved in the regulation of most key metabolic processes, including signalling [14–21], motility [22], swarming behaviour [23], and notably the promotion of tolerance to chemical stressors, among which antibiotics are naturally included [7, 11, 24, 25]. The fact that bacterial cells are excitable entities has opened many new research directions aimed at interrogating and modulating bacterial functions.

**Fig. 1:**
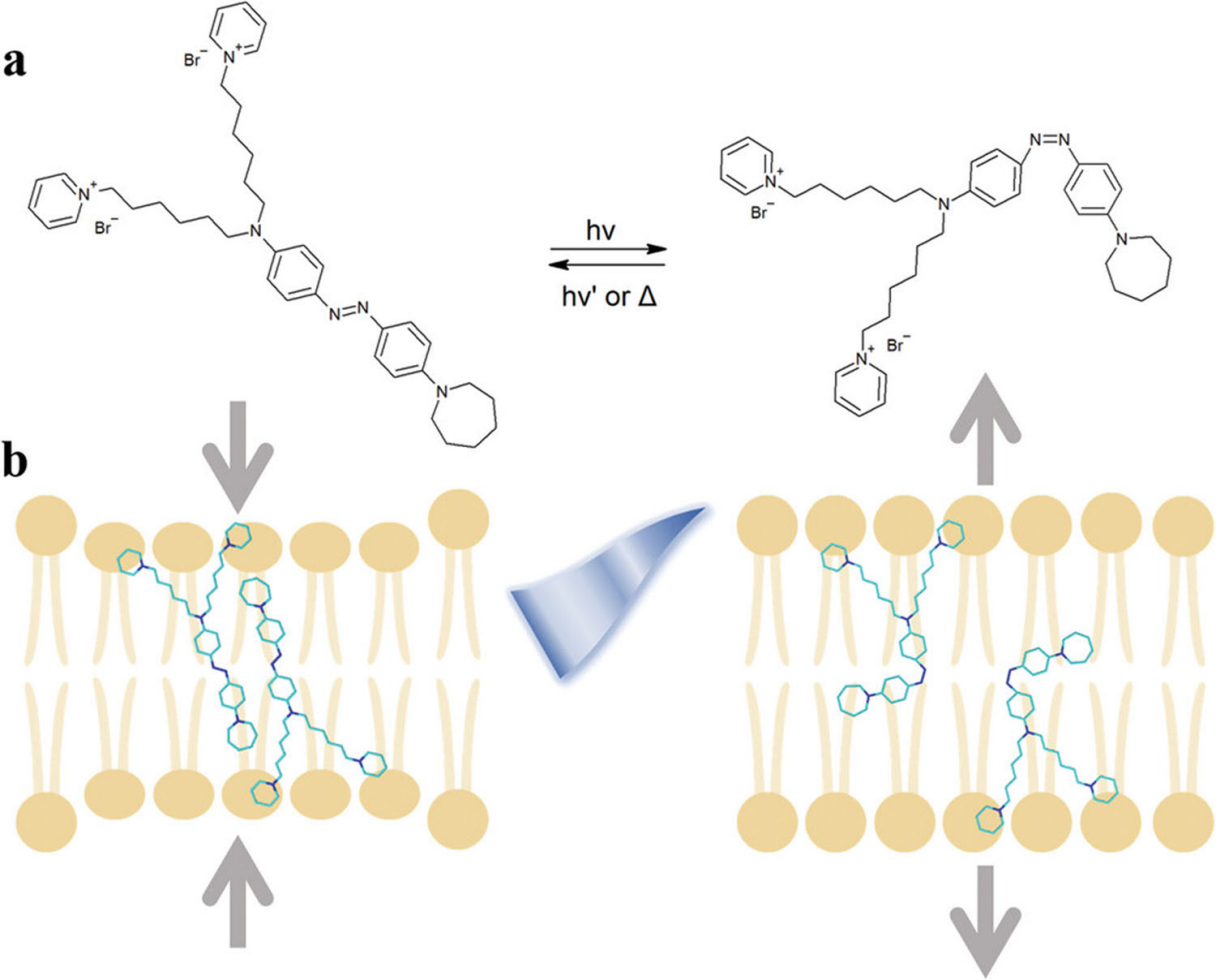
The diagram illustrates the photo-induced isomerization of Ziapin2. In the first part, the molecular structure of Ziapin2 is displayed, along with its isomerization reaction (**a**). The second part depicts the optomechanical action of Ziapin2 within the lipid membrane (**b**). When Ziapin2 is in its trans-elongated form, it can dimerize within the lipid membrane, resulting in a reduction in thickness and an increase in membrane capacitance. However, exposure to cyan light (470 nm) induces isomerization of Ziapin2 to its cis form, which breaks the dimers, leading to an increase in thickness and a decrease in membrane capacitance. Reproduced from Ref. [37] under the terms of the Creative Commons CC BY licence

Among the plethora of approaches employed to achieve this goal, the use of functional materials has proven as an effective strategy [8, 26–28]. In these regards, we have introduced the use of a membrane-targeting azobenzene photoswitch, called Ziapin2 (Fig. 1). Note, that the use of light as a spatiotemporal precise and remote tool for the purpose of bacterial excitation/interrogation can be highly advantageous, as bacteria possess a cell wall and are relatively small and motile organisms, rendering difficult the use of traditional electrical stimulation techniques [29, 30]. Ziapin2 demonstrates membrane electrical potential modulation in neurons (470 nm), both in vivo and in vitro. [31–34] Specifically, we have shown via different characterization techniques, including optical spectroscopy and neutron scattering [32, 33, 35], that in the absence of light the molecule dimerizes, inducing an increase in membrane capacitance by reducing its thickness. Following 470 nm photostimulation, the trans–cis isomerization reaction leads to dimers breaking and to the subsequent relaxation of the membrane thickness and capacitance, an effect that caused potential hyperpolarization. It is worth mentioning that the photoexcitation mechanism has been linked recently to the involvement of mechanosensitive channels [36]. Given these interesting results, we have tested this approach in bacteria, observing that the molecule triggers precise and on demand hyperpolarization spikes in *B. subtilis*, as well as photodriven potential oscillations, implying that bacteria possess a bioelectric machinery which is able to respond to transient, localized and short light stimuli [37].

Considering these results and the awareness that the efficacy of antibiotics is correlated with the cells' ability to modulate their uptake through changes in membrane potential [38], we asked ourselves whether it would be possible to modulate this phenomenon using our novel approach. Here, we take two well-known antibiotics, Kanamycin and Ampicillin, as case studies. These exhibit different mechanisms of action: while Kanamycin acts at the level of the 30S ribosomal subunit in the cytosol, inducing translation errors and leading to the production of non-functional proteins, Ampicillin targets the cell envelope by inhibiting cell wall polymerization, thus interacting with the external cellular environment. Since Kanamycin must be taken up into the cytosol whereas Ampicillin primarily acts at the cell envelope level, we expect Kanamycin to be more dependent on the membrane electrical potential than Ampicillin. We show that our data support the hypothesis that differences in molecular targeting translate into distinct impacts on antibiotic metabolism when the membrane potential is modulated.

## Results

### Photostimulation of Ziapin2 can alter population growth

**Fig. 2:**
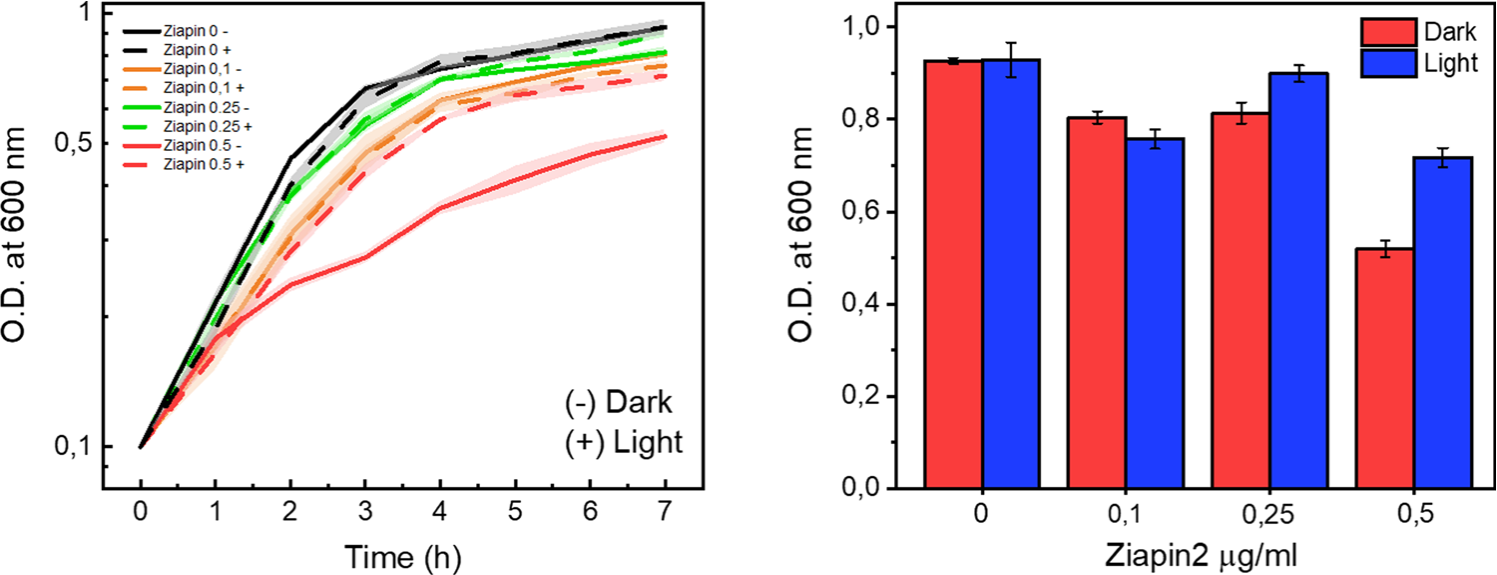
Toxicity assessment of Ziapin2. On the left, is represented the growth curve in logarithmic scale, while on the right the final Optical Density value, measured at 600 nm, reached by bacteria population at the end of the assay

The initial assessment focuses on the intrinsic toxicity of Ziapin2 towards bacterial cells (Fig. 2). We conducted an optical density variation assay to establish a threshold for Ziapin2 usage. When testing toxicity at concentrations of 0.1, 0.25, and 0.5 μg/ml, the highest concentrations showed markedly different behaviours under dark and light conditions. Specifically, while growth in dark conditions was significantly hindered due to the presence of the molecule in its trans-isomer form, recovery was observed upon light exposure. This difference can be attributed to the mechanism through which Ziapin2 enters and interacts within the phospholipid bilayer, as described by de Souza et al. [37] Based on these results, subsequent experiments were conducted using a standard concentration of 0.5 μg/ml, which was the lowest concentration at which a difference between dark and light conditions was observable without causing complete lethality to the cell population.

### Modulating Kanamycin toxicity

Initial tests were performed on Kanamycin, whose downstream effect induces the production of faulty proteins, eventually leading to metabolic and physiological insufficiency and cellular death.

Starting with kanamycin, the known threshold concentration at which the antibiotic begins to show consistent lethality is around 10 µg/ml. As the dose increases, complete lethality is achieved, culminating at 20 µg/ml. Similar to the choice of azobenzene concentrations, we standardized our assays with a sub-lethal concentration of 10 µg/ml of Kanamycin. This decision was made because using too toxic an amount of antibiotic would obscure the effect of the copresence of Ziapin2.

**Fig. 3:**
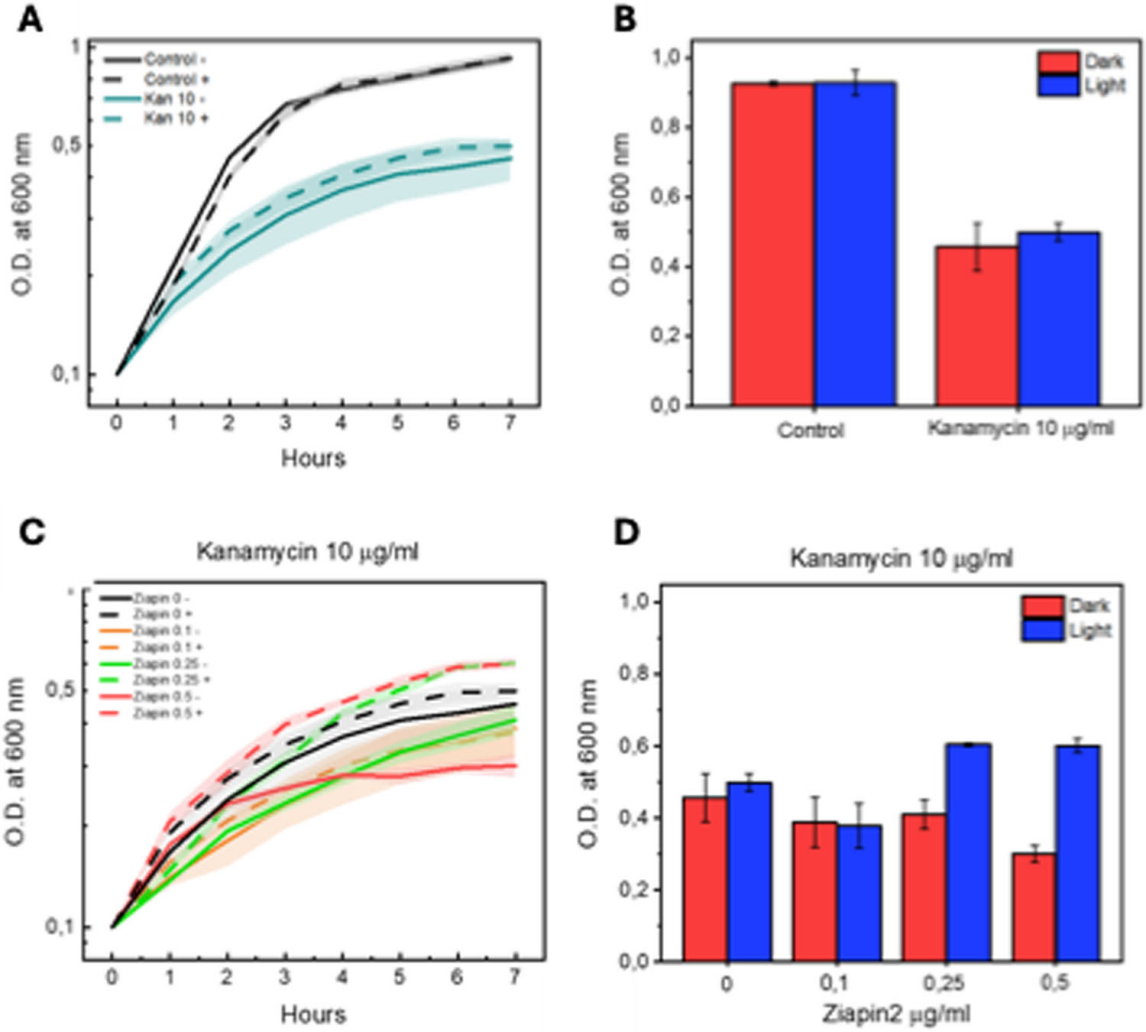
Representation of the results concerning the co-exposure of cells to Ziapin2 and Kanamycin. Figures **A** and **B** show the toxicity assessment on Kanamycin alone. Graphs **C** and **D** instead show, respectively, the growth curves of the populations studied and the final value, in terms of Optical Density, reached at the end of the experiment

Our experiments were conducted under both dark and light conditions, so it was necessary to rule out any effect of 470 nm blue light on Kanamycin's mechanism of action. We performed OD600nm variation assays, which confirmed that 470 nm light does not compromise or enhance the lethality of this antibiotic (Fig. 3). Subsequently, we conducted the same type of experiment, administering both Kanamycin and Ziapin2 at increasing concentrations, the same as those tested for lethality. As shown in Fig. 3, results indicated that the lowest concentration (0.1 μg/ml) did not show any consistent difference from the control. However, concentrations of 0.25 and 0.5 μg/ml exhibited essentially the same lethality under dark conditions, but a reliable increase in tolerance to antibiotic exposure upon 470 nm light stimulation, even surpassing the control with kanamycin-only growth rate. These initial results align with our hypothesis that membrane stretch, relaxation, and membrane potential dynamics can induce variation in the efficacy of an antibiotic with molecular targets in the cytosol, as in the case of Kanamycin.

### Modulating Ampicillin toxicity

After acknowledging the photomodulation of Kanamycin's efficacy when coupled with Ziapin2, we moved on to a radically different antibiotic, Ampicillin, that functions at the cell wall level by interfering with the polymerization of peptidoglycan. In this case, there is no internalization mechanism as shown by Kanamycin, hence, according to our initial theory, the effect induced by photostimulation in the presence of Ziapin2 should have a marginal impact on lethality.

**Fig. 4:**
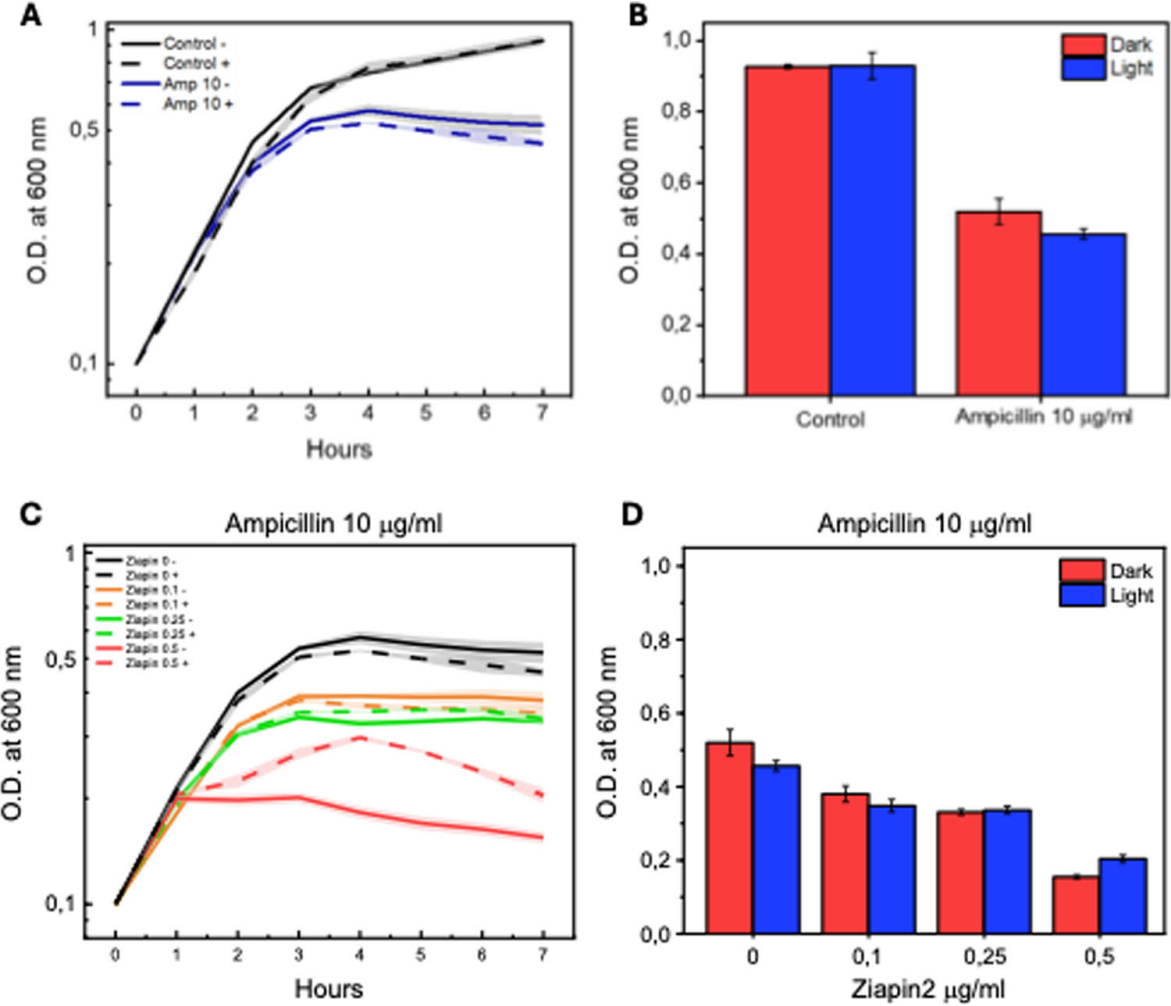
Representation of the toxicity modulation of the co-administration of Ziapin2 and Ampicillin to bacteria. **A** and **B** show the effect of Ampicillin alone, while **C** and represent, respectively, curve growth and final value of OD reached by the *B. subtilis* population

We first chose the Ampicillin standard concentration of 10 µg/ml, and assessed that photostimulation does not affect its toxicity, as shown in Fig. 4. We then focused on the effectiveness of Ziapin2 addition to the cellular suspension in co-incubation with Ampicillin. Growth assays showed that a reduced concentration of the actuator is insufficient to strongly modulate Ampicillin's activity (Fig. 4). In both dark and light conditions, adding 0.1 µg/ml and 0.25 µg/ml of Ziapin2 only resulted in a very slight, negligible increase in Ampicillin toxicity. At the highest tested concentration of Ziapin2 (0.5 µg/ml), a pronounced impact of the combined action of these two distinct molecules on the *B. subtilis* population was observed in both the presence and absence of light, leading to nearly double the lethality compared to the other samples. The rapid attainment of the plateau level, evidenced by the growth curve in Fig. 4c, is accompanied by a minimal optical density value, indicative of constrained bacterial growth. The main difference can be observed in the growth curve, as the light-exposed sample shows a slight increase in the OD peak after 4 h, followed by a decrease ending with high overall lethality. Broadly, two observations can be made from the entire essay: first, Ampicillin toxicity is strongly synergistic with Ziapin2's mechanism of action. While Ampicillin prevents cell wall formation, Ziapin2 induces strong mechanical stress on the phospholipid membrane, almost completely compromising cellular structural integrity. Secondly, as hypothesized, the lack of antibiotic molecule internalization decouples the modulation of membrane potential and antibiotic efficacy.

## Discussion and conclusions

Our experiments revealed that membrane potential dynamics has a profound influence on antibiotic efficacy. Specifically, Ziapin2, upon exposure to 470 nm light, induces a significant change in the membrane potential that directly impacts the bactericidal action of the antibiotics tested. Kanamycin, an aminoglycoside, relies on an energy-dependent uptake mechanism that is highly sensitive to membrane potential fluctuations; this dependency was evident in our results. In dark conditions, where Ziapin2-induced modulation is absent, kanamycin’s lethality remains comparable to control samples. However, when cells are exposed to 470 nm light, triggering Ziapin2’s trans-to-cis isomerization and subsequent membrane hyperpolarization, bacterial viability recovers dramatically, from 50% in non-photostimulated samples to nearly 100%, approaching the survival rate of control populations without antibiotic exposure. In stark contrast, Ampicillin, a β-lactam antibiotic that targets cell wall synthesis by inhibiting penicillin-binding proteins, operates independently of the membrane potential. Consequently, samples treated with Ampicillin did not exhibit any significant modulation in viability upon photostimulation, apart from an overall increase in lethality attributable to the combined toxic effects of Ampicillin and Ziapin2. These findings highlight that kanamycin’s efficacy is intrinsically linked to membrane potential modulation, whereas Ampicillin’s mechanism of action remains largely unaffected by such changes, consistent with our initial hypothesis.

In conclusion, this initial assessment of the possibility of modulating and controlling the interaction between bacterial cells and antibiotics using light and synthetic photoswitches, without genetic engineering, represents a first step in a completely new field of study. These preliminary yet interesting results opens to future perspectives on this complex biological phenomenon, where membrane-targeted azobenzenes will be specifically tailored to act in combination of different classes of antibiotics.

## Materials and methods

### Bacterial strains and growth conditions

Glycerol stocks of *Bacillus subtilis* NCIB 3610 wild-type strain (WT) strain (provided by Prof. Munehiro Asally’s laboratory at the University of Warwick—UK) was streaked on lysogeny-broth (LB) 1.5% agar and incubated overnight at 37 °C. A single colony was picked from this plate, inoculated in liquid LB and incubated at 37 °C shaking overnight at 160 rpm.

### Preparation of the molecules

*Ziapin2* has been synthetized by the laboratory of Prof. Chiara Bertarelli, as described in previous publications. Each stock solution was prepared by solubilizing Ziapin2 in DMSO and stocking it at room temperature and avoiding light.

### Optical density (OD) variation assays in liquid medium

Overnight culture was diluted up to OD_600 nm_ = 0.1 and, for each sample, 1 ml was moved into spectrophotometer semi-micro-cuvettes. The samples were then placed in an incubator at 37 °C, under 60rpm shaking, and exposed to λ = 470nm light pulse for 10 min every 20 min of dark. For every sample exposed to light, another one is kept in completely dark environment.

Optical density measurements were performed with *Shimadzu UV1900i* UV–Vis spectrophotometer at the wavelength of 600 nm. Data are gathered once per sample every hour for 7 h after the first point and then plotted using Origin platform.

## Data Availability

The experimental data that support the findings of this study are available in Zenodo with the identifier 10.5281/zenodo.14989767.
